# Risk factors and clinical implication of superimposed *Campylobacter jejuni* infection in patients with underlying ulcerative colitis

**DOI:** 10.1093/gastro/gov029

**Published:** 2015-07-08

**Authors:** Zubin Arora, Saurabh Mukewar, Xianrui Wu, Bo Shen

**Affiliations:** Center for Inflammatory Bowel Diseases, Digestive Disease Institute, The Cleveland Clinic Foundation, Cleveland, OH, USA

**Keywords:** *Campylobacter jejuni* infection, ulcerative colitis, risk factors, outcomes

## Abstract

**Background and aims:** Superimposed *Campylobacter jejuni* infection (CJI) has been described in patients with ulcerative colitis (UC). Its risk factors and impact on the disease course of UC are not known. Our aims were to evaluate the risk factors for CJI in UC patients and the impact of the bacterial infection on outcomes of UC.

**Methods:** Out of a total of 918 UC patients tested, 21 (2.3%) of patients were found to be positive for CJI (the study group). The control group comprised 84 age-matched UC patients who had tested negative for CJI. Risk factors for CJI and UC-related outcomes at 1 year after diagnosis of CJI were compared between the two groups.

**Results:** Ten patients (47.6%) with CJI required hospital admission at the time of diagnosis, including eight for the management of “UC flare”. Treatment with antibiotics resulted in improvement in symptoms in 13 patients (61.9%). On multivariate analysis, hospital admission in the preceding year was found to be an independent risk factor for CJI [odds ratio (OR): 3.9; 95% confidence interval (CI): 1.1–14.1] and there was a trend for chronic liver disease as a strong risk factor (OR: 5.0; 95% CI: 0.9–28.3). At 1-year follow up, there was a trend for higher rates of UC-related colectomy (28.8% *vs.* 14.3%; *P = *0.11), and mortality (9.5% *vs.* 1.2%; *P = *0.096) in the study group.

**Conclusion:** Recent hospitalization within 1 year was found to be associated with increased risk for CJI in UC patients. There was a trend for worse clinical outcomes of UC with in patients with superimposed CJI, which was frequently associated with UC flare requiring hospital admission.

## Introduction

Ulcerative colitis (UC) is a major form of inflammatory bowel diseases (IBD) characterized by chronic relapsing inflammation of the large bowel. Symptoms of UC include bloody diarrhea, abdominal pain, and urgency [1]. The pathogenesis of UC involves a complex interplay of a dysregulated immune response to an unknown environmental stimulus or dysbiosis in the colon in a genetically susceptible host [2, 3]. There is increasing evidence to suggest that the intestinal microflora may play an important role in the pathogenesis of UC [4–6]. Previous studies report an increased risk of occurrence and exacerbation of IBD following gastrointestinal (GI) infections with enteropathogenic bacteria [7–9]. Also, infection with certain GI pathogens, such as *Clostridium difficile* (*C. diff*), have been shown to be associated with worse outcomes in IBD patients [10–12].

*Campylobacter jejuni* (*C. jejuni*) is a common cause of GI infection in the United States, which is transmitted to humans through contaminated food and water [13]. Symptoms of *Campylobacter* gastroenteritis range from mild, watery diarrhea to severe dysentery [14]. The role of *Campylobacter* infection in the pathogenesis of IBD has been investigated. In a population-based cohort study with a 15-year follow-up, patients with *Campylobacter* gastroenteritis were found to be at an increased risk of developing IBD [9]. Superimposed *Campylobacter jejuni* infection (CJI) has also been frequently reported in UC patients [15–18]. However, the impact of CJI on the clinical course of UC has not been systematically studied. The aim of this study was to evaluate the risk factors for the development of CJI in UC patients and the impact of CJI on the outcome of UC.

## Patients and methods

After obtaining approval from Cleveland Clinic Institutional Review Board, we reviewed records of all UC patients who were regularly followed in the Digestive Disease Institute at the Cleveland Clinic between 2005 and 2012. The UC patients who had been tested for CJI were identified from our electronic medical records using ICD-9 codes.

## 

### Inclusion and exclusion criteria

Patients with UC with positive stool test for CJI were identified and included in the study group. These patients were compared with age-matched control UC patients with no previous history of CJI and negative stool test for CJI in a 1:4 ratio. Both inpatients and outpatients were included. Patients with Crohn’s disease (CD), microscopic colitis, and those with CJI prior to diagnosis of UC were excluded.

### Test for *C. jejuni* infection

The diagnosis of CJI was confirmed in all patients by the presence of positive testing for *C. jejuni*, defined as a positive enzyme immunoassay (EIA) as part of stool culture panel in patients with symptoms suggestive of gastroenteritis or flare-up of UC (diarrhea, abdominal pain, cramping, nausea and/or vomiting). EIA has been previously shown to be more sensitive and accurate in diagnosing CJI than the traditional culture methods [19]. The stool culture panel at our institution also routinely tests for *Salmonella, Shigella* and *Escherichia coli* O157:H7 by stool culture.

### Clinical variables

Retrospective chart review was performed by one investigator (Z.A.) to extract relevant clinical and demographic information, including age, gender, disease extent and severity, clinical symptoms, risk factors for CJI, and antibiotics used for the treatment of CJI. The anatomical extent of colitis (proctitis, left-sided colitis or extensive colitis), UC medications [5-aminosalicylic acid (5-ASA), corticosteroids, immunomodulators (azathioprine, 6-mercaptopurine, and methotrexate), anti-tumor necrosis factor (TNF) biologics], non-steroidal anti-inflammatory drugs (NSAID) use and presence of any extra-intestinal manifestations were also abstracted.

In addition, we recorded information regarding UC-related emergency room (ER) visits that did not result in hospitalization, UC-related hospitalization for medical management, and number and type of UC-related surgeries performed at our institution in the subsequent one year following testing for CJI. Severity of UC activity at the time of testing for CJI was assessed, based on patients’ most recent endoscopic findings. The last colonoscopy performed at our institution prior to testing for CJI was reviewed for this purpose. Endoscopic disease severity was scored according to the Mayo Endoscopic Score (0 = normal, 1 = mild, 2 = moderate and 3 = severe) [20].

### Outcome variables

The primary outcome of the study was poor clinical outcome of UC, as defined by the occurrence of one or more of the following: UC-related ER visit, hospitalization, surgery, or death at 1 year following *C. jejuni* testing.

### Statistical analysis

Descriptive statistics were computed for all variables. These included means and standard deviations or medians and interquartile ranges (IQR) for continuous variables, and frequencies for categorical variables. Comparisons between groups were made by using the two-tailed *t*-test (or Wilcoxon rank sum test, as appropriate) for continuous variables, and the chi-squared test (or the Fisher exact test, as appropriate) for categorical variables. In addition, multivariate logistic regression analyses were performed to evaluate factors associated with CJI, as well as the risk for the poor UC outcome. All statistical analyses were performed with SPSS software version 16 (SPSS, Chicago, IL, USA). A *P-*value less than 0.05 was considered statistically significant.

## Results

A total of 918 UC patients had been tested at our institution during the period studied. Out of these, 21 patients (2.3%) were found to be positive for CJI. These patients were included in the Study group. The Control group comprised of 84 age-matched UC patients who tested negative for CJI. These were selected in a random manner from the remaining 897 UC patients who had been tested for CJI. Stool specimens of all patients in the Study and Control groups were negative for other enteric pathogens routinely tested for as part of the stool culture panel. The median duration between most recent endoscopic examination and CJI status evaluation was 3 days (IQR: 0, 29). The median duration of follow-up was 4.8 years (IQR: 2.3, 6.8).

### Comparison of demographic and clinical variables

A summary of the demographic and clinical characteristics of patients with and without CJI is given in Table 1. On univariate analysis, there were no significant differences between the Study and Control groups in terms of tobacco use, NSAID use, extra-intestinal manifestations, recent overseas travel or antibiotic use within the previous month, or severity of disease activity prior to CJI testing.

**Table 1.** Demographics and clinical characteristics

Characteristics	Study group (*n = *21)	Control group (*n = *84)	*P*-value
Male patients, *n* (%)	9 (42.9)	48 (57.1)	0.24
Age at CJI testing, years	46.9 ± 14.6	42.0 ± 17.3	0.24
Duration of UC, years	8.8 (2.9–18.4)	3.5 (1.4–9.6)	**0.04**
Inpatient status at CJI testing, *n* (%)	10 (47.6)	26 (31.0)	0.20
UC extensive colitis, *n* (%)	10 (52.6)	53 (67.1)	0.24
Extra-intestinal manifestations, *n* (%)	1 (5.9)	13 (16.5)	0.45
Past history of chronic liver disease, *n* (%)	4 (19.0)	3 (3.6)	**0.03**
Past history of PSC	2 (9.5)	0 (0.0)	**0.04**
Chronic NSAID use, *n* (%)	4 (19.0)	20 (25.0)	0.78
Tobacco use, *n* (%)	10 (47.6)	37 (44.6)	0.80
Family history of IBD, *n *(%)	3 (14.3)	10 (12.0)	0.72
Past history of *C. diff* infection, *n* (%)	2 (9.5)	1 (1.2)	0.10
Past history of CMV colitis, *n* (%)	0 (0.0)	1 (1.2)	0.99
Past history of CJI colitis, *n* (%)	1 (4.8)	0 (0.0)	0.20
Recent antibiotic use, *n* (%)	4 (19.0)	7 (8.3)	0.22
Recent overseas travel, *n* (%)	2 (9.5)	1 (1.2)	0.10
Hospitalization within last 12 months, *n* (%)	6 (28.6)	8 (9.5)	**0.03**
Severity of UC on recent endoscopy, *n* (%)			0.07
Normal	1 (7.1)	1 (1.3)	
Mild	4 (28.6)	14 (18.7)	
Moderate	2 (14.3)	35 (46.7)	
Severe	7 (50.0)	25 (33.3)	
Baseline 5-ASA use, *n* (%)	14 (66.7)	55 (66.3)	0.97
Baseline corticosteroid use, *n* (%)	8 (38.1)	28 (33.7)	0.71
Baseline immunomodulator use, *n* (%)	4 (19.0)	12 (14.5)	0.74
Baseline biologics use, *n* (%)	3 (14.3)	5 (6.0)	0.20

Continuous values presented as mean ± standard deviation or medians (interquartile ranges). Bold values signifies statistical significance when *P*-value is < 0.05.

ASA = aminosalicylic acid; *C. Diff = Clostridium difficile*; CJI = *Campylobacter jejuni* infection; CMV = cytomegalovirus; IBD = inflammatory bowel disease; NSAID = non-steroidal anti-inflammatory drug; PSC = primary sclerosing cholangitis; UC = ulcerative colitis.

### Risk factors for CJI

Univariate analysis showed that patients in the Study group were more likely to have a longer duration of UC (8.8 *vs.* 3.5 yrs; *P* *=* 0.04), were more likely to have concurrent chronic liver disease (19.0% *vs.* 3.6%; *P* *=* 0.03) including primary sclerosing cholangitis (PSC) (9.5% *vs.* 0%; *P* *=* 0.04) and were more likely than those in the Control group to have been admitted to the hospital in the previous 12 months (28.6% *vs.* 9.5%; *P* *=* 0.03). Exposures to proton pump inhibitors and H_2_ receptor antagonists were similar in both groups (23.8% *vs.* 21.4%; *P* *=* 0.77).

Of the four patients with chronic liver disease in the Study group, two patients had cirrhosis secondary to PSC, one had cirrhosis secondary to non-alcoholic steatohepatitis (NASH), and one had autoimmune hepatitis without evidence of cirrhosis on imaging. Of the three patients with chronic liver disease in the Control group, one had cirrhosis secondary to hepatitis C virus infection, one had cirrhosis secondary to NASH, and one had chronic hepatitis C virus infection without evidence of cirrhosis on imaging.

On multivariate analysis, hospital admission in the preceding 12 months was found to be an independent risk factor for CJI (odds ratio [OR]: 3.9; 95% confidence intervals [CI]: 1.1–14.1). The presence of chronic liver disease also seemed to confer significant risk for CJI (OR: 5.0; 95% CI: 0.9–28.3), although this did not reach statistical significance (Table 2).

**Table 2.** Multivariate analysis of risk factors associated with *Campylobacter jejuni* infection.

Variable	Odds ratio	95% CI	*P*-value
Duration of UC, every 5-year increase	1.2	1.0–1.5	0.09
Presence of chronic liver disease	5.0	0.9–28.3	0.07
Hospitalization in the last 12 months	3.9	1.1–14.1	**0.04**

Bold values signifies statistical significance when *P*-value is < 0.05.

CI = confidence interval; UC = ulcerative colitis.

### Management of superimposed CJI

Ten patients (47.6%) with CJI required hospital admission at the time of diagnosis, including eight for the management of “UC flare”. Treatment regimens for CJI included oral erythromycin or azithromycin in 18 patients (85.7%) and oral ciprofloxacin or levofloxacin in the remaining three (14.3%). Treatment with antibiotics resulted in improvement in symptoms in 13 patients (61.9%), while eight (38.1%) continued to experience persistent symptoms. None of the patients tested *C. diff*-positive following treatment with antibiotics.

### Risk factors for poor clinical outcome

The differences in the clinical outcomes of patients with and without CJI are summarized in Table 3. At 1-year follow-up after CJI testing, rates of UC-related ER visit (9.5% *vs.* 3.6%; *P* *=* 0.25), hospitalization (14.3% *vs.* 16.7%; *P* *=* 0.99), surgery (28.8% *vs.* 14.3%; *P* *=* 0.11) and mortality (9.5% *vs.* 1.2%; *P* *=* 0.096) were not statistically different between patients in the Study and Control groups.

**Table 3.** Ulcerative colitis outcomes at 1 year after diagnosis

Characteristics	Study group (*n = *21)	Control group (*n = *84)	*P*-value
UC-related ER visit, *n* (%)	2 (9.5)	3 (3.6)	0.25
UC-related hospitalization, *n* (%)	3 (14.3)	14 (16.7)	1.00
UC-related surgery, *n* (%)	6 (28.8)	12 (14.3)	0.11
UC-related mortality, *n* (%)	2 (9.5)	1 (1.2)	0.096

ER = emergency room; UC = ulcerative colitis

Univariate analysis was performed, of the risk factors associated with poor UC outcomes (Table 4). A greater number of patients with poor outcomes had severe disease on recent endoscopy than those with good outcomes (57.1% *vs.* 29.4%; *P* *=* 0.009). Inpatient status at CJI testing (64.3% *vs.* 23.4%; *P* < 0.001) and baseline corticosteroid use (51.9% vs. 28.6%; *P* *=* 0.036) were found to be associated with poor outcomes.

**Table 4.** Univariate analysis of risk factors associated with 1-year poor outcomes

Characteristics	Good outcomes (*n = *77)	Poor outcomes (*n = *28)	*P*-value
Male patients, *n* (%)	41 (53.2)	16 (57.1)	0.72
Age at CJI testing, years	42.8 ± 17.2	43.2 ± 16.1	0.91
Patients with CJI, *n* (%)	14 (18.2)	7 (25.0)	0.44
Duration of UC	6.2 (1.8–10.8)	2.6 (1.3–13.9)	0.45
Inpatient status at CJI testing	18 (23.4)	18 (64.3)	**<0.001**
UC extensive colitis, *n* (%)	43 (59.7)	20 (76.9)	0.15
Extra-intestinal manifestations, *n* (%)	11 (15.3)	3 (12.5)	1.00
Chronic NSAID use, *n* (%)	20 (27.4)	4 (14.3)	0.17
Tobacco use, *n* (%)	38 (50.0)	9 (32.1)	0.11
Family history of IBD, *n* (%)	11 (14.5)	2 (7.1)	0.51
Past history of chronic liver disease, *n* (%)	6 (7.8)	1 (3.6)	0.67
Past history of PSC	1 (1.3)	1 (3.6)	0.46
Past history of *C. diff* infection, *n* (%)	2 (2.6)	1 (3.6)	1.00
Past history of CMV colitis, *n* (%)	1 (1.3)	0 (0.0)	1.00
Past history of CJI colitis, *n* (%)	0 (0.0)	1 (3.6)	0.27
Recent hospitalization in last 3 months, *n* (%)	10 (13.0)	5 (17.9)	0.54
Recent antibiotic use, *n* (%)	8 (10.4)	3 (10.7)	1.00
Recent overseas travel, *n* (%)	2 (2.6)	1 (3.6)	1.00
Severity of UC on recent endoscopy, *n* (%)			**0.009**
Normal	2 (2.9)	0 (0.0)	
Mild	18 (26.5)	0 (0.0)	
Moderate	28 (41.2)	9 (42.9)	
Severe	20 (29.4)	12 (57.1)	
Baseline 5-ASA use, *n* (%)	52 (67.5)	17 (63.0)	0.67
Baseline corticosteroid use, *n* (%)	22 (28.6)	14 (51.9)	**0.036**
Baseline immunomodulator use, *n* (%)	14 (18.2)	2 (7.4)	0.23
Baseline biologics use, *n* (%)	4 (5.2)	4 (14.8)	0.20

Continuous values presented as mean ± standard deviation or medians (interquartile ranges). Bold values signifies statistical significance when *P*-value is < 0.05.

ASA = aminosalicylic acid; *C. diff = Clostridium difficile*; CJI = *Campylobacter jejuni* infection; CMV = cytomegalovirus; IBD = inflammatory bowel disease; NSAID = non-steroidal anti-inflammatory drug; PSC = primary sclerosing cholangitis; UC = ulcerative colitis.

On multivariate analysis, inpatient status at presentation was found to be an independent risk factor for poor outcomes (OR: 5.7; 95% CI: 1.6–20.3) (Table 5).

**Table 5.** Multivariate analysis of risk factors associated with 1-year poor outcomes

Variable	Odds ratio	95% CI	*P*-value
*Campylobacter jejuni* infection	0.6	0.1–2.9	0.56
Inpatient status at presentation	5.7	1.6–20.3	**0.007**
Severe UC on recent endoscopy	1.3	0.4–4.6	0.69

Poor outcomes include UC-related emergency room visit, hospitalization, surgery or death. Bold values signifies statistical significance when *P*-value is < 0.05.

CI = confidence interval; UC = ulcerative colitis.

## Discussion

GI superinfections are commonly seen in IBD patients and cause significant morbidity [16, 17, 21, 22]. *C. jejuni* is a common cause of GI infection in IBD patients and has even been implicated in the pathogenesis of IBD [6, 9]. The present study attempted to assess the risk factors for CJI in UC patients and its effects on the clinical outcomes of UC. The frequency of CJI in this cohort of UC patients was 2.3%. We found that hospital admission in the preceding 12 months and presence of chronic liver diseases were risk factors for CJI in UC patients. Nearly half of the patients with CJI needed to be admitted to the hospital at the time of diagnosis for management of their symptoms and there was a trend for worse clinical outcomes in patients with CJI.

Previous studies have shown that CJI leads to disruption of the intestinal barrier function and increased epithelial permeability [23, 24]. This is associated with increased translocation of intestinal microflora, which may act as stimuli for inflammation in a susceptible host, thus suggesting a mechanism by which CJI may contribute to the pathogenesis of IBD [24–26]. This suggestion is supported by data from a murine model of UC, in which CJI was shown to increase the severity of colitis [27]. In addition, epidemiological cohort studies have demonstrated an increased risk of development and/or exacerbation of symptoms of IBD after CJI [7, 9, 18, 28]. Consistent with those reports, nearly 50% of patients in our study needed to be admitted to the hospital at the time of diagnosis of CJI, most of them due to a flare-up of their UC symptoms. In these patients, it is important to identify the presence of superimposed CJI, since the symptoms can mimic that of UC flare. Appropriate treatment with antibiotics can result in the eradication of infection and improvement in symptoms.

In our series of UC patients, the rate of CJI was found to be 2.3%. This rate is similar to two earlier studies, in which the rates of CJI were 3/113 patients (2.6%) and 5/237 patients (2.1%) [17, 21]. Patients hospitalized in the previous 12 months were observed to be at increased risk for CJI. Additionally, the presence of chronic liver disease also appeared to be a strong risk factor for CJI in UC patients, although this did not reach statistical significance. This association of CJI with chronic liver disease is novel and has not previously been described in clinical studies. In mouse models, the liver has been shown to play a major role in clearing *C. jejuni* from the bloodstream and a strong influx of immune cells into mouse liver has been demonstrated with CJI [29, 30]. In fact, chronic liver disease has also been shown to increase the risk for other gut infections, including *C. diff* [31]. It is possible that interruption of similar immunological mechanisms in the presence of chronic liver disease in UC patients leads to increased risk for development of CJI. This remains to be further explored.

Currently, there are conflicting data regarding clinical outcomes following GI infections in IBD patients. While multiple studies have demonstrated worse clinical outcomes in IBD patients following *C. diff* infection [10–12], this has not been demonstrated for other enteropathogenic bacteria, including *C. jejuni* [16]. In our study, there was a trend for worse clinical outcomes—such as UC-related surgery (28.8% *vs.* 14.3%) and UC-related mortality (9.5% *vs.* 1.2%)—in CJI patients than in controls. Although these did not reach statistical significance, they are direct outcome measures suggesting aggressive disease in CJI patients. Additionally, inpatient status at the time of CJI was an independent risk factor for worse outcomes and, given that nearly half of the patients with CJI needed to be admitted to the hospital at presentation, this confers a significant risk for poor outcomes.

It is possible that CJI alters the natural course of UC by activating abnormal mucosal immune response, thus leading to more inflammation and more severe disease that result in worse outcomes. Another possibility is that sicker patients may be more susceptible to developing CJI, which would put them at higher risk for worse outcomes. This idea is supported by our findings, that patients with worse outcomes were more likely to be on corticosteroids at baseline and have severe disease on endoscopy.

Our findings have several clinical implications: for UC patients who present with a disease flare, especially those with concurrent liver diseases, the possibility of a superimposed CJI should always be kept in mind. Although the effect of treatment for CJI on UC outcomes has not been assessed, we routinely treat any patients with CJI with appropriate antibiotics. The eradication of CJI may not merely mitigate symptoms but can potentially eradicate the pathogenic risk factors for exacerbation of UC-related inflammation as a result of CJI. Aggressive treatment of UC may also be warranted in patients with CJI, given the association of CJI with higher severity of UC. Finally, these patients may require closer monitoring and more frequent follow-up for early recognition of complications related to their severe disease.

The present study has some limitations. Our findings are based on a cohort of 21 CJI patients, which somewhat limits the value of the findings and raises the possibility of the study being underpowered. However, balanced against this is the fact that CJI infection is relatively uncommon and the cohort presented is thus one of the largest to date. Our study population was being followed-up at a tertiary referral IBD center; this might have introduced a referral bias. We only recorded the ER visits and hospitalization at our own institution and could have missed interim events at other hospitals. As this was a retrospective study, we were unable to record information regarding the dietary risk factors for *C. jejuni*, the strain of *C. jejuni* and document the eradication of *C. jejuni* after treatment. We used the recent endoscopic examination prior to CJI for the assessment of disease severity. This might have been a source of bias, given the fluctuating nature of the disease course, because of changes in disease severity between the times of endoscopic evaluation and CJI status assessment. However, the delay time between endoscopic examination and CJI status evaluation was very small, thus minimizing the effects of this measurement bias. Also, since the sample size and number of patients with chronic liver disease was small, meaningful comparisons based on cause of liver disease, presence and stage of cirrhosis were not possible.

In conclusion, recent hospitalization within 12 months and maybe the presence of chronic liver diseases were found to be associated with increased risk for CJI in UC patients. There was a trend for worse clinical outcomes of UC in patients with superimposed with CJI.

## Funding

The research and education activity of Bo Shen, MD, is supported by the Ed and Joey Story Endowed Chair.

*Conflict of interest statement*: none declared.
